# The Effects of Intermittent Fasting during the Month of Ramadan in Chronic Haemodialysis Patients in a Tropical Climate Country

**DOI:** 10.1371/journal.pone.0114262

**Published:** 2014-12-29

**Authors:** Wan Ahmad Hafiz Wan Md Adnan, Nur Lisa Zaharan, Mun Hoe Wong, Soo Kun Lim

**Affiliations:** 1 Nephrology Unit, Department of Medicine, Faculty of Medicine, University Malaya, Lembah Pantai, 50603, Kuala Lumpur, Malaysia; 2 Department of Pharmacology, Faculty of Medicine, University Malaya, Lembah Pantai, 50603, Kuala Lumpur, Malaysia; University of Sao Paulo Medical School, Brazil

## Abstract

**Background:**

Chronic kidney disease is an emerging problem in the majority Muslim countries. Despite the uncertainties of the risks involved, some Muslim patients undergoing chronic haemodialysis choose to observe intermittent fasting during the month of Ramadan. This study aims to investigate the effect of Ramadan fasting in haemodialysis patients residing in a tropical climate country.

**Methods:**

This prospective cross sectional study recruited Muslim patients on regular haemodialysis from three haemodialysis centres in Kuala Lumpur from 15^th^ July 2011 to 29^th^ August 2011. Patients who fasted for any number of days were included (n = 35, 54% female, age 54±11 years). 89% of patients fasted for more than 15 days and 49% were diabetics. Dialysis parameters and blood samples were obtained one week prior to Ramadan and during the last week of Ramadan. The differences in dialysis parameters and biochemical values pre- and end-Ramadan were examined using paired t-test.

**Results:**

Both pre- and post-dialysis weight were significantly decreased during Ramadan fasting compared to the month prior (*p* = <0.001). There was a significant decrease in the amount of ultrafiltration (*p* = 0.002). There were no significant differences in dry weight, inter-dialytic weight gain, mean urea reduction ratio or blood pressure measurements comparing pre- and end of Ramadan fasting. There was a significant increase in serum albumin level (*p* = 0.006) and decrease in serum phosphate level (*p* = 0.02) at the end of Ramadan.

**Conclusion:**

Ramadan fasting is associated with reduced weight, improved serum albumin and phosphate level in our population of haemodialysis patients. A larger multi-centre study will allow us to understand more about the effects of fasting in this population.

## Introduction

The Muslim population makes up approximately 23% of the world population corresponding to 2.1 billion people [1]. Amongst the pillars of Islam that Muslims adhere to is to fast during Ramadan, the ninth month in the Islamic lunar calendar. Muslims observed fasting from dawn until sunset, during which no food or drinks are allowed to be consumed. After sunset, they may eat and drink without any restrictions. Those who are sick, pregnant or nursing women, menstruating women and those who undertake a journey are amongst those exempted from this act of faith.

Although the Quran specifically mentioned permissibility for the sick to abstain from fasting, the Epidemiology of Diabetes and Ramadan 1422/2001 (EPIDIAR) study (n =  12,243) involving 13 countries has observed that many Muslims with diabetes (43% of type 1 and 79% of type 2 diabetes patients) still chose to fast during Ramadan [2]. Extrapolating from the EPIDIAR results, it was estimated that some 50 million Muslims with diabetes fast during Ramadan worldwide [3]. Recognizing the importance of this issue, the American Diabetes Association (ADA) has taken the initiative to publish expert recommendations on the management of the Muslim diabetes patients who fast during Ramadan [3].

Chronic kidney disease (CKD) is an emerging problem in the majority Muslim countries such as the Arab countries (including Saudi Arabia, Egypt, and United Arab Emirates) and others such Indonesia, Turkey and Malaysia [4]–[7]. However, there is a lack of epidemiological data on the prevalence of CKD in many of these countries [4], [5]. The increasing prevalence of diabetes in these countries will also contribute to increased prevalence of CKD in years to come [4]. Despite the uncertainty of the risks involved in CKD patients undergoing haemodialysis, some Muslim patients with CKD still voluntarily observed Ramadan fasting. There is a lack of clinical guideline on how best to manage these patients.

It was shown previously that the urinary volume, electrolytes, pH and nitrogen excretion from urine samples of healthy individuals with normal homoeostatic mechanisms remained within the physiological limits during Ramadan fasting [8]–[9]. Small studies in the cohort of patients with chronic kidney problems especially in post-transplant patients suggested that Ramadan fasting is quite safe in these patients [10]–[15]. However, there is paucity of data on Ramadan fasting in CKD patients on chronic haemodialysis. This prospective cross sectional study aims to investigate the effect of voluntary Ramadan fasting in a cohort of haemodialysis patients in a tropical climate country with Muslim as majority.

## Subjects and Methods

### Study design and study population

This prospective cohort study was performed in three haemodialysis centres in Kuala Lumpur from 15^th^ July 2011 to 29^th^ August 2011. Ethical approval for this study was obtained from the Ethics committee of the University Malaya Medical Centre.

Prior to the Ramadan month of 2011, all Muslim patients on regular haemodialysis treatment of at least three times per week were identified from the participating haemodialysis centres (n = 70). Information regarding the study was given by a clinician. Participation was on voluntary basis. Written informed consent which has been approved by the ethical committee was obtained from all participants. The participants were allowed to break their Ramadan fasting at any time of the day for any reasons. Muslim patients who have the intention to fast during Ramadan for any number of days were recruited (n = 35, 54% female, age 54±11 years). The baseline characteristics of these patients are presented in Table 1. 89% of these patients fasted for more than 15 days. The duration of Ramadan fasting was 14 hours and the average temperature during the month was 32 degree Celsius.

**Table 1:** Characteristics participants in the effects of voluntary Ramadan fasting in haemodialysis patient study.

Patient characteristics	All patients	Non-diabetes patients	Diabetes patients only
	(n = 35) (n, %)	(n = 18) (n, %)	(n = 17) (n,%)
Age (mean year, SD)	54±11	52±14	56±6
Gender: Female	19 (54%)	9 (50%)	10 (58%)
Co-morbidity			
Diabetes	17 (49%)	-	-
Hypertension	31 (89%)	15 (83%)	16 (94%)
IHD	7 (20%)	3 (17%)	4 (24%)
Duration on haemodialysis	6.4±4.9	8.5±5.1	4.1 ±3.6
(mean year, SD)			
Causes of end-stage kidney disease			
Diabetes	15 (43%)	-	15 (88%)
Hypertension	11 (31%)	10 (55%)	1 (6%)
Polycystic kidney disease	1 (3%)	1 (6%)	0
Systemic lupus erythematosus	1 (3%)	1 (6%)	0
Unknown	7 (20%)	6 (33%)	1 (6%)
Type of vascular access			
AV fistula	32 (91%)	16 (89%)	16 (94%)
Cuffed catheter	3 (9%)	2 (11%)	1 (6%)
Duration of fasting (days, SD)	24±7	26±6	22±8

### Data collection

#### Dialysis parameters

The dialysis parameters for the last six sessions of haemodialysis prior to the beginning of Ramadan 2011 were collected from individual patients. The parameters collected include the documented dry weight, the actual pre- and post- dialysis weight, inter-dialytic weight gain (IDWG), total ultrafiltration per dialysis session and both systolic and diastolic blood pressure pre- and post-dialysis. A second set of dialysis parameters were collected from the six consecutive haemodialysis sessions in the last two weeks of Ramadan.

#### Biochemical parameters

A mid-week pre-dialysis biochemical profiles were collected from participants in the last week prior to the beginning of Ramadan 2011 as a baseline. A similar random biochemical profiles were collected from the same patients in the last week of Ramadan 2011, as a reflection of Ramadan fasting. These biochemical profiles included renal profile (sodium, potassium, urea and serum creatinine), bone profile (albumin, calcium and phosphate), haemoglobin, fructosamine, HbA1c and lipid profile (total cholesterol, triglycerides, HDL- and LDL-cholesterol). Urea reduction ratio (URR) was calculated using pre- and post- dialysis urea values. The post-dialysis urea was taken immediately after completion of haemodialysis.

#### Clinical parameters

Hypotensive episodes were defined as any recorded episodes of systolic blood pressure below 100 mmHg during dialysis session. The data were collected from six consecutive dialysis sessions prior to the beginning of Ramadan and the last week of Ramadan. Self-reported symptomatic hypoglycaemic events were recorded by the attending clinician. The hypoglycaemic symptoms included shakiness, dizziness, hunger, nausea and feeling faints or blackouts.

### Statistical analysis

Means and standard deviations were calculated for continuous variables. The differences in dialysis parameters and biochemical values pre- and end Ramadan were examined using paired t-test. In addition, separate analysis were performed in subgroup of patients with diabetes (n = 17) and non-diabetes (n = 18). Statistical analyses were performed using SPSS version 21 (SPSS, Inc., Chicago, Illinois). *p*<0.05 is considered as statistically significant.

## Results

### Dialysis parameters

Both pre- and post-dialysis weight were significantly decreased during Ramadan fasting compared to the month prior as presented in Table 2. The reduction in pre- and post-dialysis weight were more marked in the non-diabetes group. There were significant decreases in the amount of ultrafiltration both in diabetes and non-diabetes group. Overall, there were no significant changes in dry weight, inter-dialytic weight gain, or blood pressure measurements comparing pre- and end of Ramadan fasting. However, in the non-diabetes group, post haemodialysis systolic and diastolic blood pressure measurements significantly increased at the end of Ramadan fasting.

**Table 2:** Dialysis parameters pre- and end Ramadan fasting in haemodialysis patients (mean ± SD) and the *p*-values of changes (End Ramadan- pre-Ramadan values).

Parameters	Patients groups	Pre-Ramadhan	End of Ramadhan	*p*-value
Dry Weight (kg)	All patient	65.2±12.3	65.2±12.5	NS
	Non-diabetes	63.2±12.7	62.8±12.9	NS
	Diabetes	67.4±11.9	67.4±11.9	NS
Pre HD Weight (kg)	All patient	67.5±13.0	66.7±13.2	<0.001
	Non-diabetes	65.1±13.3	63.9±13.4	<0.001
	Diabetes	70.1±12.6	69.7±12.6	NS
Post HD Weight (kg)	All patient	65.5±12.4	64.7±12.7	<0.001
	Non-diabetes	63.2±12.8	62.1±13.1	<0.001
	Diabetes	67.8±12.0	67.5±12.0	0.01
IDWG (kg)	All patient	2.1±0.7	2.0±0.8	NS
	Non-diabetes	2.0±0.7	1.7±0.7	NS
	Diabetes	2.2±0.7	2.2±0.9	NS
Ultrafiltration (L)	All patient	2.2±0.7	1.9±0.8	0.002
	Non-diabetes	2.6±0.9	2.1±0.7	0.009
	Diabetes	2.4±0.6	2.2±0.8	0.02
Pre HD SBP (mmHg)	All patient	148±19	149±17	NS
	Non-diabetes	141±17	144±18	NS
	Diabetes	155±19	153±16	NS
Post HD SBP (mmHg)	All patient	130±19	132±19	NS
	Non-diabetes	123±18	130±20	0.02
	Diabetes	136±19	134±19	NS
Pre HD DBP	All patient	78±9	79±9	NS
	Non-diabetes	79±9	79±10	NS
	Diabetes	78±9	79±7	NS
Post HD DBP (mmHg)	All patient	71±10	72±10	NS
	Non-diabetes	69±11	72±12	0.01
	Diabetes	72±10	71±8	NS

NS *non-significant.*

### Biochemical parameters

Overall, there was a significant increase in serum albumin level (*p* = 0.006) and a significant decrease in serum phosphate level (*p* = 0.02) at the end of Ramadan compared to pre-Ramadan (Table 3). There was a significant increase in the level of calcium (*p* = 0.03) and a significant decrease in the level of phosphate (*p*<0.0001) in the non-diabetes group during Ramadan fasting which were not observed in the diabetes group. In the subgroup of patients with diabetes, a significant reduction in haemoglobin and HDL cholesterol values were observed at the end of Ramadan fasting (*p* = 0.01). The level of fructosamine, as marker of short term glucose control was not significantly different during Ramadan fasting in patients with diabetes.

**Table 3:** Biochemical parameters pre- and end Ramadan fasting in haemodialysis patients (mean ± SD) and the *p*-values of changes (End-Ramadan - pre Ramadan values).

Parameters	Patient Groups	Pre- Ramadhan	End of Ramadhan	*p*-value
Urea Reduction Ratio	All patients	67.2±7.8	66.5±8.0	NS
	Non-diabetes	69.6±6.8	68.4±7.5	NS
	Diabetes	66.6±8.4	64.4±8.3	NS
Sodium (mmol/L)	All patients	135±3	135±3	NS
	Non-diabetes	136±3	136±2	NS
	Diabetes	134±2	134±3	NS
Potassium (mmol/L)	All patients	4.9±1.2	4.8±1.2	NS
	Non-diabetes	4.6±0.6	4.5±0.8	NS
	Diabetes	5.2±1.5	5.2±1.4	NS
Urea (mmol/L)	All patients	18.0± 3.8	16.8±4.2	0.05
	Non-diabetes	17.5±4.3	16.1±4.9	NS
	Diabetes	18.6±3.1	17.6±3.0	NS
Creatinine (µmol/L)	All patients	925±200	916±237	NS
	Non-diabetes	971±180	978±241	NS
	Diabetes	867±209	847±220	NS
Haemoglobin (g/dL)	All patients	11.6±1.9	11.3±1.6	NS
	Non-diabetes	11.4±1.6	11.5±1.5	NS
	Diabetes	11.7±2.2	11.1±1.8	0.01
Albumin (g/L)	All patients	33±3	34±4	0.006
	Non-diabetes	33±4	35±4	0.01
	Diabetes	33±3	33±3	NS
Corrected Calcium (mmol/L)	All patients	2.41±0.22	2.47±0.27	0.05
	Non-diabetes	2.45±0.19	2.56±0.26	0.03
	Diabetes	2.36±0.23	2.36±0.24	NS
Phosphate (mmol/L)	All patients	1.8±0.5	1.7±0.5	0.02
	Non-diabetes	1.8±0.4	1.4±0.5	<0.0001
	Diabetes	1.9±0.5	1.9±0.4	NS
Fructosamine (µmol)	All patients	306±116	322±95	NS
	Non-diabetes	260±100	289±80	NS
	Diabetes	356±107	361±95	NS
HbA1c (%)	All patients	7.2±2.2	7.2±2.0	NS
	Non-diabetes	5.6±0.6	5.7±0.6	0.02
	Diabetes	8.9±1.8	8.9±1.8	NS
Total Cholesterol (mmol/L)	All patients	4.5±1.0	4.4±0.8	NS
	Non-diabetes	6.4±9.9	4.3±0.8	NS
	Diabetes	4.8±1.0	4.6±0.9	NS
LDL-cholesterol (mmol/L)	All patients	2.5±0.9	2.5±0.8	NS
	Non-diabetes	2.3±0.8	2.4±0.8	NS
	Diabetes	2.8±0.8	2.7±0.9	NS
HDL-cholesterol (mmol/L)	All patients	1.0±0.3	1.0±0.4	NS
	Non-diabetes	1.1±0.4	1.2±0.4	NS
	Diabetes	0.9±0.3	0.8±0.2	0.01

NS *non-significant.*

### Clinical parameters

**Figure 1:**
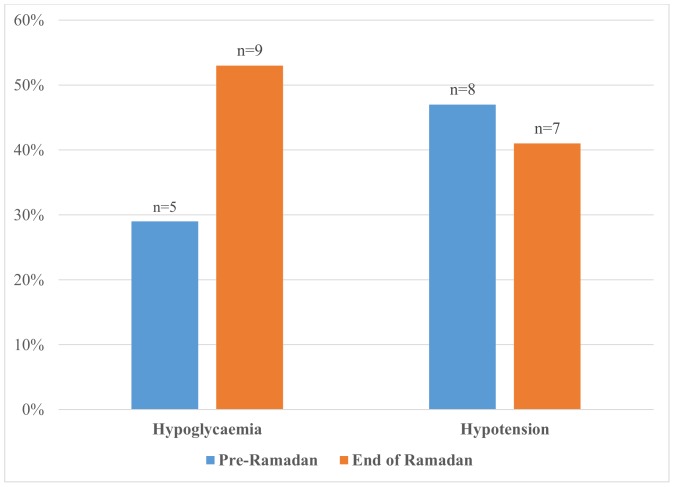
Percentage of observed hypotensive episodes during dialysis (systolic blood pressure <100 mmHg) and self-reported hypoglycaemic episodes in patients with diabetes pre- and end of Ramadan fasting.

Overall, there was a reduction in the number of hypotensive episodes during dialysis session with 45% (n = 17) of patients experiencing at least one episode of systolic blood pressure of <100 mmHg pre-Ramadan and 35% (n = 11) at the end of Ramadan. None of the non-diabetic patients reported hypoglycaemic episodes. In the subgroup with diabetes, there was an increase in the hypoglycaemic episodes as reported by the patients (Fig. 1).

## Discussion

All patients who participated in this study tolerated Ramadan fasting quite well. This is similar with other studies on Ramadan fasting in patients with pre-dialysis CKD [13]–[14]. There was a significant decrease in the weight of patients who voluntary fasted during Ramadan, both pre- and post- dialysis especially in the non-diabetes group. However, the inter-dialytic weight gain remained stable comparing pre- and end of Ramadan. The reduction in body weight is consistent with other studies on Ramadan fasting both in healthy adults and in CKD patients [13]–[14], [17]. The reduction in body weight may be explained by the reduction in both fluid and caloric intake during the month of Ramadan. It is interesting to note that there was a significant reduction in post-dialysis weight during Ramadan (mean 64.7 kg), but not the dry weight (mean 65.2 kg). In contrast, Rashed *et al* noted that patients on long term haemodialysis who fasted during Ramadan may experience an increase in body weight and fluid overload between dialysis sessions due to the tendency of these patients to increase their food consumption at nights of Ramadan [16], [18]. In different cultures, different kinds of foods are consumed during Ramadan. Some prefer festive foods with higher sugar, protein and fat content while others simply reduced their food intake with resultant differential effects on body weight [19].

This current study did not find significant changes in renal profiles comparing pre- and end Ramadan values. In contrast, a study on pre-dialysis CKD patients in Egypt and another on long term haemodialysis patients in Saudi demonstrated an increase in serum potassium levels during Ramadan fasting [10], [14]. The increase in the serum potassium was attributed to the types of food consumed during Ramadan which are rich in potassium such as huge amount of dates, apricot and figs early in the morning and during breaking of fast in these two countries [14]. These types of food are not as popular in Malaysia. In the post renal transplant population with normal allograft function, studies have also shown that there were no harmful effects of Ramadan fasting in terms of urinary and serum biochemical markers [12], [18]

There was a significant increase in serum albumin level and a decrease in serum phosphate level at the end of Ramadan. This was more marked in the non-diabetes patients. El-Wakil *et al* demonstrated that there was a significant reduction in serum albumin levels pre- and post- Ramadan comparing between 15 pre-dialysis CKD patients and 6 healthy controls in Egypt [14]. The results of our study may be explained by reduction of both fluid and food intake during Ramadan fasting. Reduction of water intake may induce higher level of albumin [20]. We feel that the higher albumin level does not reflect higher protein consumption, especially when the calcium levels was significantly increased in the non-diabetes group. Higher serum calcium can be found in patients with dehydration as well. This is consistent with the reduction of pre- and post-dialysis weight observed in this group during Ramadan fasting. We only compared the biochemical values pre- and end Ramadan in haemodialysis patients and therefore comparisons were not made with healthy controls.

Overall, there were no significant changes in cholesterol level pre and at the end of Ramadan fasting. However, in the subgroup of diabetes, a significant decrease in the HDL- cholesterol value was observed. Inconsistent findings have been observed on the effect of Ramadan fasting on cholesterol levels, especially the HDL cholesterol [17], [21]–[22]. This may relate to changes in the caloric intake during Ramadan in this subgroup of patients.

There were no significant changes in both systolic and diastolic blood pressure pre-dialysis in our cohort of patient pre- and end Ramadan. This is consistent with the findings from Perk *et al* in which it was demonstrated that Ramadan fasting was not associated with significant changes in ambulatory blood pressure in treated hypertensive patients [23]. Similar findings were also observed in the cohort of pre-dialysis CKD patients [13]–[14]. In addition, we did not observe significant increase in the hypotensive episodes during Ramadan fasting. Thus we can assume that Ramadan fasting is safe and in terms of blood pressure control.

The HbA1c and fructosamine were not significantly different pre- and at the end of Ramadan in this study. In terms of self-reported hypoglycaemic event, although an increased number of hypoglycaemic episodes were observed especially in patients with diabetes, this trend is not significant. This is probably due to the small number of patients involved. Under reporting of hypoglycaemic episodes may also be a possibility. The EPIDIAR study has shown that although the overall incidence of hypoglycaemic episodes were rather low, diabetic patients experience more frequent episodes of severe hypoglycaemia during Ramadan fasting compared to prior [2].

### Strength and limitations

This study examined an issue that is of growing importance in Muslim patients who undergo dialysis. The data from the EPIDIAR study has shown the medical professions that Muslim patients do observe Ramadan fasting voluntarily even without being advised by their health practitioners [2]. Due to the nature of Ramadan fasting, it will be difficult to perform a randomized controlled trial for researchers and clinicians to be able to reach a more definite conclusion. Our cross sectional study seek to bridge the gap of knowledge on the effect of Ramadan in chronic haemodialysis patients in a tropical country.

Our study recruited patients from 3 different haemodialysis centres, in which the total number of patients exceeds 150. However, given the demographic of the study centres chosen, there were only 70 Muslim patients, in which only half agreed to participate in this study (n = 35, 17 diabetics). The small sample size limits the generalizability of our findings in haemodialysis population and require replication study with larger sample size, if possible inclusive of patients from other countries who observe Ramadan fasting. There may be selection bias as the patients who volunteer to participate in this study may be of better health status compared to those who decline to participate and those who did not observe Ramadan fasting.

This study is performed in a tropical country. The Muslim populations are spread over the world with most are concentrated in the Arab countries and the African region [1]. The weather in these other countries can be very extreme and thus the level of hydration may differ. In healthy adults, it was shown that the fluid turnover rates and fluid intake were markedly different between those who observed their Ramadan fasting in Malaysia [9] and those in Sudan [24]. The effect of Ramadan fasting in patients on haemodialysis may also vary with seasonal variations. As Ramadan follows the lunar calendar, people living in countries with four season's climate may have to observe Ramadan fasting at different seasons over the years. During summer, the duration of Ramadan fasting will also be longer depending on the time of sunrise and sunset and thus may have different effects on the parameters in the patients involved. We do not have access to dietary and physical activity information from the patients which may explain some of the variation in the parameters observed. As this study was designed to be non-interventional, we did not advice patients to change the dosing or timing of their medications during Ramadan, although they may have received the advice from other clinicians. The change in medications, especially anti-diabetic agents may account for the reduction in weight as observed.

Given that our patient population were already on regular haemodialysis, we did not examine the tubular functions of these patients during Ramadan fasting. Using urinary NAG as a marker of renal tubular injury, El-Wakil et al showed that there was a slight reduction in renal tubular functions in patients with CKD compared to normal controls [14]. Reversible renal tubular dysfunction was also observed in a study by Cheah *et al* on healthy adults with no permanent renal damage [8]. In our haemodialysis practice, we do not routinely measure the residual amount of urine output in chronic haemodialysis patients. Residual renal clearance in haemodialysis patient has been shown to be an important predictor of survival in haemodialysis patient [25].

### Implications/recommendations

There is a lack of study that specifically addressed CKD and dialysis management during Ramadan. There is also a lack of epidemiological data available on the proportion of CKD patients, pre- or on dialysis who fast during Ramadan. The proportion of Muslim patients with CKD, with or without dialysis, who fast and what effect does Ramadan fasting have in these patients needs to be examined. A large multi-country study, like the EPIDIAR study [2], need to be undertaken in the cohort of Muslim patients with CKD. A recommendation for Ramadan fasting in patients on peritoneal dialysis was recently published [26]. It is high time for renal physicians to recognize this issue and come together, like the ADA [3] to form expert opinion on how best to manage these patients.

## Conclusions

Ramadan fasting is associated with reduced weight and improved serum albumin and phosphate levels in our small sample of chronic haemodialysis patients residing in a tropical climate country. Larger and more inclusive studies need to be undertaken to assess the clinical effects of Ramadan fasting to provide practitioners with better understanding of this religious rituals in chronic haemodialysis patients.
